# Bone Morphogenetic Protein as Bone Additive around Dental Implant and its Impact on Osseointegration: a Systematic Review

**DOI:** 10.30476/DENTJODS.2021.90931.1536

**Published:** 2022-09

**Authors:** Fathima Banu Raza, Sivakumar Vijayaragavalu, Anand Kumar Vaidyanathan

**Affiliations:** 1 Dept. of Prosthodontics, Faculty of Dental Sciences, Sri Ramachandra Institute of Higher Education and Research (SRIHER 00 116, Tamil Nadu, India; 2 Dept. of Life Sciences(Zoology) Manipur University, Imphal 795003, Manipur, India; 3 Dept. of Prosthodontics, Faculty of Dental Sciences, Sri Ramachandra Institute of Higher Education and Research (SRIHER 00 116, Tamil Nadu, India

**Keywords:** Bone Morphogenetic Protein 2, Drug carrier, Bone-Implant inter-face

## Abstract

**Statement of the Problem:**

Bone morphogenetic protein (BMP), a potential osteoinductive agent, was systematically reviewed for merits and demerits when used as a bone additive that was intervened during the surgical phase of dental implant placement; and suitable drug carriers that could withstand the functional load and deliver BMP at its lowest concentration.

**Purpose:**

To identify the carriers and concentration of BMP acceptable during surgical phase of implant placement and evaluate its efficacy in bone gain and osseointegration.

**Materials and Method:**

The study design was systematic review. Literature search as per PICO format was carried out within a time range from 2000 to July 2021. The review fol-lowed PRISMA guidelines and registered with the PROSPERO (CRD42020171667). The focus question included the population with an intra-oral implant placed in both animal and human models that were intervened with BMP-2 as an external additive biomaterial during the surgical phase. 2631 articles selected from the initial search were systematically filtered and yielded 16 articles that were qualitatively analysed

**Results:**

The inter-rater reliability and level of agreement were 93.71%, κ(Kappa)>0.81 re-spectively. Results revealed the collagen carrier was commonly used for BMP delivery but lacked the property to withstand functional load and sustained release. BMP concentration varied in the range of 0.215μg to 0.8mg and the study revealed significantly indifferent out-come with low dose compared to the highest dose. BMP supplement showed better osseointe-gration in comparison with non-supplemented sites during the early period (within 6 months).

**Conclusion:**

BMP at lower concentrations and with appropriate carriers, collagen sponge, hydroxyapatite/tricalcium phosphate (HA/TCP) with a bio ceramic bulking agent, and poly (D, L-lactide-co-glycolic acid) (PLGA) reinforced with gelatin/HA/TCP accelerated bone growth during the initial stages of healing. Further long-term clinical trials for dental implant, analysing the sustained release of BMP with biodegradable and load-bearing carriers should be considered.

## Introduction

Long-term survival of implant requires balanced bone remodelling to maintain the bone architecture and osseointegration [ 1 - 2 ]. Bone remodelling occurs by the process of resorption and deposition of bone around the implant, and it begins from the early phase of implant healing [ 3 ]. Osteotomy followed by torquing of the implant in the bone initiates osteoclastic differentiation to remove the dead bone and debris by releasing fibroblast growth factor-2, interleukin-1, interleukin-6 and macrophage colony-stimulating factor [ 4 ]. The osteoclastic activity is followed by the release of growth factors like insulin-like growth factor, platelet-rich fibrin, bone mor-phogen-etic protein (BMP) to promote vascularization and osteogenesis to remodel the peri-implant bone [ 5 ]. The imbalance in the release of bone growth promo-ting cells alters bone remodelling and accelerates the bone loss around dental implants. Hence, the addition of bone growth-promoting factors or bone additives will fasten the bone formation by promoting osteogenesis [ 6 - 7 ]. 

Grafting with growth-promoting factors or bone substitute materials regenerates the bone through either osteo-induction or osteo-conduction [ 6 ]. The primary choice of bone substitutes were autogenous grafts as it has the ability of osteo-induction, which is differentiation of immature cells to osteoblast [ 7 ]. However, due to limitations in harvesting the autogenous grafts, allografts, xenografts, and alloplasts came into existence [ 6 , 8 ]. Allografts and xenografts have varied host cell acceptance while, the alloplastic graft has only osteo conductive potential (acts as a scaffold) [ 9 ]. Hence, alternative materials were searched for the achievement of osteo-induction without host rejection of the graft that promotes bone cell migration, differentiation, and proliferation to enable bone remodelling [ 10 ]. 

BMP are growth factors, belonging to the transforming growth factor-β family, induces new bone formation by inducing differentiation of multi-potent cells [ 11 - 12 ]. BMPs were first identified when demineralized lyophilized bone was incorporated in ectopic site induced bone formation, and in 1971, Urist [ 13 ] addressed the role of BMP’s in osteo-induction. Further research succeeded in cloning the genes, which code for BMP [ 14 ]. BMP forms about 1 part per billion of the bone, and were also isolated from demineralized bone matrix, *Escherichia coli*, osteosarcoma cell line [ 13 , 15 - 16 ]. Though 20 types of BMP were identified, BMP-2, 7, 8 and 9 were proved effective in enhancing osteogenic activity; and BMP 2 and 7 are approved for human use [ 14 , 17 - 18 ]. 

The influence of BMP in wound healing, repair, and new bone formation is most widely researched in the field of orthopaedics [ 19 ]. In dentistry, the literature reveals that BMP is widely used as a bone augmentation material or an implant surface modifier [ 20 - 21 ]. BMP was delivered as a bone growth additive during the surgical phase of dental implant placement would accelerate the bone remodelling [ 22 ]. BMP per se is efficient in bone regeneration but the use of carriers helps in local delivery and also reduces the concentration of BMP required for its action in the grafted site [ 23 ]. Carriers retain the BMP for a longer duration and allow a sustained release to ensure healing and regeneration completion [ 24 ]. The carriers are selected based on their biodegradability and osteo-inductivity [ 23 - 24 ]. Though the efficacy of BMP in bone regeneration is evident, its effect around the implant region is unclear. Implant commonly made of titanium has the potential to alter cell infiltration. In addition, bone adjoining the dental implant receives functional load and the carrier utilized should have sufficient strength to withstand the force [ 25 ]. Hence, a literature search was carried to identify an appropriate carrier and concentration of BMP that would be effective around the dental implant for bone regeneration during surgical placement. The review involved both animal and human models to identify the newer trends developed in animals, which is still a void in a human clinical trial. This systematic review exclusively evaluated the types of carriers and concentration of BMP in promoting osseointegration when used as a bone growth additive during the surgical phase of implant treatment.

## Materials and Method

The systematic review was conducted in accordance with Preferred Reporting Items for Systematic Reviews and Meta-Analyses (PRISMA updated October 2015) guidelines and was registered with the PROSPERO international prospective registry for systematic reviews (CRD42020171667). The focus question was established as per the Population, Intervention, Comparison, Outcome format (PICO). Population of the research being intra-oral implant placed in an edentulous site and intervened with BMP. The BMP was comparatively evaluated with a non-BMP group for varied concentration and delivery methods. The outcome of the systematic review was summarised based on the osseointegration.

### Eligibility Criteria

Two independent researchers performed the electronic search using Medical Subject Heading (MeSH) keywords for PICO format with a Boolean index of AND between the components. Population involved partially edentulous; Intervention was Bone morphogenetic protein-2 (BMP-2) in various concentrations, carriers; Comparison was no intervention; the Outcome evaluated was an improvement in bone formation and osseointegration. The MeSH keywords for population component were the oral cavity OR edentulous jaw OR jaw, edentulous, partially OR mandible OR alveolus, dental OR jaw OR implant, dental OR immediate dental implant loading OR early dental implant loading that included human and animal model. MeSH keywords for the intervention were Bone morphogenetic protein OR BMP 2 protein, human. The above-mentioned intervention was compared with the keywords; BMP delivery vehicle OR BMP carrier OR BMP concentration OR BMP dose frequency. Finally, the outcome variable evaluated included crestal bone OR peri implantitis OR mucositis, oral OR bone defect OR osseointegration OR implant survival. The keywords ((((Bone Morphogenetic Protein OR BMP 2 OR BMP)) AND (Dental Implant OR Alveolar ridge OR Edentulous Arch)) AND (Crestal bone loss OR Peri-implantitis OR Osseointegration)) AND (BMP carrier OR BMP concentration OR) were used in various combinations to extract the relevant articles.

### Information sources

Literature published in the time range of January 2000 to July 2021 was sought after by three independent researchers in the following databases (search engines): Medline (Pubmed), Elsevier (Science Direct), Cochrane (Cochrane library), IndMED, and Embase (OVID). The search for grey literature was carried out in the Opengray database. 

### Inclusion criteria

The eligibility for inclusion of the articles in the review was considered only when the BMP was used as a bone additive along with surgical placement of an implant in the oral cavity. Only prospective studies involving animal and human models with a minimum follow-up period of six months were included. Implant placement in any other region was not considered in the systematic review. In addition, the review did not include data when BMP was used without placement of the implant or as an implant surface modifier. Experimental comparative study design with intervention for animal experiments was included in the review. To evaluate the highest level of evidence in human research, randomized controlled trials were included. This was conducted to analyse the deficiency in human research and the scope of future research in a clinical trial.

### Data collection

The data were extracted by the first author (FB) and filled into a pre-defined form that evaluated the basic characteristics of the study: authors, year of publish, study design, aim and outcome. The data extracted were tabulated chronologically and the data synthesis was based on evidence tables and descriptive summaries (Table 1, Table 2). The second author (SK) checked the information collected, and the third author (AK) settled the disagreement between the authors.

**Table 1:** Descriptive of the study design and summary

Author & Year	Study Design	Aim	Comparison	Outcome
Fiorellini JP *et al.*, 2001	Randomized split-mouth design	Evaluated the effect of recombinant human bone morphogenetic protein-2 (rhBMP-2) on early bone formation within the perforations of dental implants in beagle dogs	rhBMP-2 in a methylcellulose gel vs methylcellulose gel alone	rhBMP-2 increased the rate and extent of bone formation in combination with dental implants
Matin K *et al.* 2003	Comparative study	Evaluated the bone regeneration of recombinant human bone morphogenetic protein-2 (rhBMP-2) around immediate implants placed in maxillary sockets in rats	rhBMP-2 in PLGA –coated gelatin sponge around immediate implants vs PLGA-Gelatine sponge alone	Presence of rhBMP-2 facilitated the regeneration of bone around immediate implants
Jung RE, *et al.*, 2003	RCT	Evaluated the impact of adding recombinant human bone morphogenetic protein-2 (rhBMP-2) to a xenogeneic bone substitute mineral (Bio-Oss) on guided bone regeneration therapy	rhBMP-2 vs non- rhBMP-2 implant	rhBMP-2 has the potential to improve and accelerate guided bone regeneration therapy
Jung RE, *et al.*, 2003	RCT	Evaluated the long-term outcome of implants placed with a xenogeneic bone substitute material and a collagen membrane with or without the addition of rhBMP-2	rhBMP-2 vs non- rhBMP-2 implant	No statistically significant differences were observed between the test and control sites after 3 and 5 years
Smeeds R *et al.*, 2009	Comparative study	Compared the recombinant human bone morphogenic protein 2 (rhBMP-2) in the healing of large buccal alveolar defects during osseointegration of trans gingivally inserted implants	rhBMP-2 in Calcium phosphate vs non-rhBMP-2 calcium phosphate	The amounts of rhBMP-2 utilised did not enhance implant osseointegration in large buccal defects compared to the control site
Jaebum Lee, *et al.*, 2010	Comparative study	Compared the effect of implants soaked in or coronally coated with rhBMP-2 on new bone formation and resident bone remodelling	Soak loaded vs Coronal loaded rhBMP-2	Coronal-load implants of rhBMP-2 appears to be a viable technology to support local bone formation and osseointegration
Huh, *et al.*, 2010	Comparative study	Effect of Escherichia coli–derived recombinant human BMP-2 (ErhBMP-2) coated onto anodized implant to stimulate osseointegration and the vertical augmentation of the alveolar ridge	ErhBMP-2 (0.75 vs 1.5mg/ml) coated vs uncoated implant	The ErhBMP-2 coating on an anodized implant significantly increased implant stability on completely healed alveolar ridges
Lu SX, *et al.*, 2013	Comparative study	Evaluated the local bone formation and osseointegration following surgical implantation of rhBMP-2 in a compression resistant matrix	ACS carrier rhBMP-2 vs HA/β-TCP/collagen rhBMP-2	In comparison with the ACS, compression resistant matrix significantly enhanced rhBMP-2-induced bone formation and osseointegration
Chang, *et al.*, 2017	Comparative study	Evaluated the PLGA microspheres encapsulating BMP-2 within a gelatin/HA/β-TCP cryogel composite in supra-alveolar ridge augmentation	PLGA microspheres in HA/ β-TCP BMP-2 vs HA/ β-TCP BMP-2 vs HA/ β-TCP vs Uncoated implant	The gelatin/HA/β-TCP cryogel composite with PLGA microspheres encapsulating BMP-2 facilitated supra-alveolar ridge augmentation
Ragheb, *et al.*, 2017	RCT	Evaluated and compared the implant stability and crestal bone height of titanium dental implants with and without rhBMP in immediately loaded implant supported mandibular overdenture	rhBMP-2 HA/TCP coated vs Uncoated implants	Conventional titanium dental implant coated with ready-made BMP may induce less crestal bone resorption and better implant stability when compared with conventional titanium dental implant in immediately loaded trials
Schom *et al.*, 2017	Comparative study	Evaluated the generation of vertical bone growth with disc-shaped collagenous scaffold containing rhBMP-2 and VEGF placed around the coronal part of the implant	non-rhBMP-2 vs non-rhBMP-2 in ICBM VS rhBMP-2 in ICBM vs rhBMP-2 in ICBM with VEGF.	Combination of rhBMP-2 and VEGF applied locally by using a collagenous carrier improved vertical bone generation
Sun YK, *et al.*, 2018	Comparative study	Effect of collagen membrane) soaked with rhBMP-2 for the treatment of peri-implant dehiscence defects	Collagen membrane rhBMP-2 vs collagen membrane non-BMP vs Uncoated implant	The use of rhBMP-2 soaked on Collagen Membrane as a carrier material did not result in superior bone formation compared to control sites without rhBMP-2. However, the use of fixation pins to stabilize the Collagen Membranes did exert a positive effect on peri-implant bone regeneration
Lyu HZ *et al.*, 2020	Comparative study	Evaluated the efficacy of rhBMP-2 loaded hydrogel composite for bone formation around dental implant in minipig mandible bone defect models	Non-rhBMP-2 in HA/β-TCP microspheres vs rhBMP-2 in HA/β-TCP microspheres	rhBMP-2 loaded hydrogel composite promoted osteogenesis around dental implant in bone defect, and enhanced osseointegration.
Tan MH *et al.*, 2020	Comparative study	Investigated bone regeneration in a three-wall defect during immediate implant placement, using porous biphasic calcium phosphate granules with or without recombinant Human rhBMP-2.	Non-rhBMP-2 in vs rhBMP-2 in biphasic calcium phosphate with collagen membrane	rhBMP-2 resulted in significant new bone formation in a non-contained defect around an immediate implant
Chao YL *et al.*, 2021	Comparative study	Investigated the potential of the BMP-2 peptide combined with HA/β-TCP/collagen composite in comparison with rhBMP-2 in repairing a peri-implant critical size defect	rhBMP-2 peptide in HA/β-TCP/Collagen at various concentration vs rhBMP-2 in HA/β-TCP /Collagen	BMP-2 peptide at 20 mg/mL had similar osteoinductive performance to the rhBMP-2 at 0.02 mg/ml.
Chao YL *et al.*, 2021	Comparative study	Investigate the potential of low-dose rhBMP-2 combined with HA/β-TCP/ Collagen composite in repairing the peri-implant critical size defect	Non-rhBMP-2 in HA/β-TCP/Collagen vs rhBMP-2 at various concentration in HA/β-TCP/Collagen	HA/TCP/Collagen with 50µg rhBMP-2 manifested strong osteogenic potential with better implant stability

rhBMP-recombinant Bone Morphogenetic Protein; PLGA- poly (D,L-lactide-co-glycolic acid); ErhBMP-2-Escherichia coli–derived recombinant human BMP-2; ACS-Absorbable Collagen Sponge; HA/β-TCP- Hydroxyapatite beta Tricalcium phosphate; ICBM-Insoluble Collagen Bone Matrix; VEGF- Vascular Endothelial Growth Factor

**Table 2:** Descriptive summary of Materials and Methodology used in the studies

Author Name & Year	Type of Sample	No. of Samples	No. of Implants	Comparison	Follow up Period	Type of Carrier	Quantity (Concentration) of BMP	Method of Investigation	Measuring Outcome
Fiorellini JP *et al.*, 2001	Female beagle dogs	2	19	Control: 0.9% methylcellulose gel Test: 0.16 mg/mL rhBMP-2 in 0.9% methylcellulose gel	4 weeks	0.9% methylcellulose gel	0.16 mg/mL	Histometric analysis	Bone formation
Matin K *et al.* 2003	Male Wistar rats	8	16	Control: no grafting materials Test 1: (PLGA) –coated gelatin sponge Test 2: rhBMP-2 with PLGA –coated gelatin sponge	90-day	PLGA –coated gelatin sponge	Not reported	Scanning electron microscopy	Bone regeneration
								Contact microradiography	Bone-implant contact
								Confocal laser microscopy	Bone implant contact
Jung RE, *et al.*, 2003	Human	11	34	Control: Xenogeneic bone substitute Test: Xenogeneic bone substitute with rhBMP2	0 & 6 months	Xenogeneic Bone Substitute & collagen membrane	0.5mg (1 ml of 0.5 mg/ml) rhBMP-2	Clinical	Vertical Bone Height
								Histologic	Type of Bone
								Histometric	Bone Area Density & Mineralisation
Jung RE, *et al.*, 2008	Human	11	34	Control: Xenogeneic bone substitute Test: Xenogeneic bone substitute with rhBMP2	0, 36 & 60 months	Xenogeneic Bone Substitute & collagen membrane	0.5mg (1 ml of 0.5 mg/ml) rhBMP-2	Vas Score	Gingival condition
								Clinical Examination	Gingival probing depth
								Radiographic	Marginal bone loss
Smeeds R *et al.*, 2009	Labrador/golden retriever cross-bred dogs.	6	24	Control: Calcium Phosphate Test: rhBMP-2 in Calcium phosphate carrier	4 months	Calcium phosphate	0.3 µg	Clinical Examination	Implant failure
								Histology	Bone formation
								Histometric	Bone Gain, Bone Implant contact, Osseo-integration
Jaebum Lee, *et al.*, 2010	Hound Labrador Mongrel dogs	12	72	Test 1-Soak loaded rhBMP-2 Test 2- Coronal loaded rhBMP-2	8 weeks	-	30 µg rhBMP-2	Radiographic	Seroma Formation
								Histological	Type of bone
								Histometric	Bone Height, Area, Density & Bone implant contact
Huh, *et al.*, 2010	Male adult Beagle dogs	6	36	Control: uncoated implant- Test 1: ErhBMP-2 (0.75 mg/mL concentration)- Test 2: ErhBMP-2 (1.5 mg/mL concentration)	4 & 8 weeks	-	10µg (0.75 mg/ml) and 20µg (1.5 mg/ml)- ErhBMP-2	Radiographic	Bone growth & Bone loss
								Resonance Frequency analyser (Ostell Mentor)	Implant Stability
								Surgical Measurement	Bone deposition
Lu SX, *et al.*, 2013	Adult male Hound Labrador Mongrel dogs	5	30	Control: ACS carrier rhBMP-2- Test: compression resistance matrix (collagen/β-TCP/hydroxyapatite) rhBMP-2	4 & 8 weeks	ACS & compression resistance matrix	0.8 mg rhBMP-2	Clinical,	Healing soft tissue
								Radiograph,	Seroma formation
								Histologic	Seroma
								Histometric	Bone Area, Density & Bone-implant contact
Chang, *et al.*, 2017	Male Sprague Dawley rats	16	32	Control: uncoated implant- Test 1: HA/ β-TCP- Test 2: HA/ β-TCP BMP-2 - Test 3: HA/ β-TCP BMP-2 in PLGA microspheres	4 weeks	HA/ β-TCP & HA/ β-TCP in PLGA	3.2µg (0.16 µg/ul in 20µl) & 0.215 µg BMP-2	Micro Computed Tomography	Relative bone volume
								Scanning Electron Microscope & Histology.	Osteogenesis, Mineralisation
Ragheb, *et al.*, 2017	Human	10	20	Control: non-BMP- Test-rhBMP-2 HA/ β-TCP	0, 6 & 12 months	HA/ β-TCP	0.25 mg vial rhBMP-2	Radiograph	Bone height & Bone loss
								RFA analyser (Ostell Mentor)	Implant Stability
Schom *et al.*, 2017	Mini pigs	12	72	Control 1: no intervention- Control 2: ICBM- Test 1: ICBM containing rhBMP-2- Test 2: ICBM containing rhBMP-2 and Vascular Endothelial Growth Factor (VEGF)	2, 4 and 12 weeks	ICBM	138 μg rhBMP-2 and 18.4 μg VEGF	Histologic	Bone formation
								Histomorphometry	Bone-implant contact, Bone-volume-density, Bone gain
Sun YK, *et al.*, 2018	Mongrel dogs	5	15	Control- Uncoated implant- Test 1- collagen membrane without BMP- Test 2 – collagen membrane rhBMP-2	4 weeks	Collagenated Synthetic Bone & Collagen Membrane	0.1mg (0.2 ml of 0.5 mg/ml) rhBMP-2	Micro Computed Tomography &	Total augmented volume
								Histomorphometry analysis	Bone implant contact
Lyu HZ *et al.*, 2020	Yucatan male minipigs	5	20	Control: No intervention- Test 1: HA/ β-TCP microspheres- Test 2: rhBMP-2 loaded in HA/ β-TCP microspheres	4 weeks	HA/β-TCP microspheres	300 μg of rhBMP-2.	Plain radiographs	Bone loss
								Micro-CT	Bone volume, Trabecular formation
								Histological evaluation	Bone-to-implant area and contact ratios
Tan MH *et al.*, 2020	Female adult minipigs	5	5	Control: Biphasic Calcium Phosphate granules covered with collagen membrane- Test: rhBMP-2 in porous Biphasic calcium phosphate granules covered with collagen membrane	6 months	HA/TCP (porous BCP granules)	0.43 mg rhBMP-2 /cc of defect size	Histologic evaluation	Bone remodelling, Bone regeneration and presence of any inflammatory reaction
Tan MH *et al.*, 2020	Female adult minipigs	5	5	Control: Biphasic Calcium Phosphate granules covered with collagen membrane- Test: rhBMP-2 in porous Biphasic calcium phosphate granules covered with collagen membrane	6 months	HA/TCP (porous BCP granules)	0.43 mg rhBMP-2 /cc of defect size	Histologic evaluation	Bone remodelling, Bone regeneration and presence of any inflammatory reaction
								Histomorphometry	Bone-to-implant contact, Bone area
Chao YL *et al.*, 2021	Male beagle dogs	4	24	Control: HA/ β-TCP/ Collagen composite- Test 1: HA/ β-TCP/ Collagen with 1 mg BMP-2 peptide- Test 2: HA/ β-TCP/ Collagen with 5 mg BMP-2 peptide- Test 3: HA/ β-TCP/ Collagen with 50 µg rhBMP-2	4 and 8 weeks	HA/ β-TCP/Collagen composite	50 µg rhBMP-2	Resonance frequency analysis	Implant stability
								Radiographic and Micro-CT	Bone Defect fill
								Histomorphometry	New bone formation
Chao YL *et al.*, 2021	Male Beagle dogs	5	50	Control: no intervention, Test 1: HA/ β-TCP/Collagen composite,Test 2: HA/ β-TCP/Collagen +5µg rhBMP-2,Test 3: HA/ β-TCP/Collagen +20µg rhBMP-2, Test 4: HA/ β-TCP/Collagen +50 µg rhBMP-2.	4 and 8 weeks	HA/ β-TCP/Collagen composite	rhBMP-2 solution at 5, 20, or 50 µg.	Resonance frequency analysis	implant stability
								Radiographic and Micro-CT	Bone Defect fill
								Histomorphometry	New bone formation, Bone density

rhBMP-recombinant Bone Morphogenetic Protein; PLGA- poly (D,L-lactide-co-glycolic acid); ErhBMP-2-Escherichia coli–derived recombinant human BMP-2; ACS-Absorbable Collagen Sponge; HA/β-TCP- Hydroxyapatite beta Tricalcium phosphate; ICBM-Insoluble Collagen Bone Matrix; VEGF- Vascular Endothelial Growth Factor

### Risk of bias assessment

The “systematic review centre for laboratory animal experimentation (SYRCLE) RoB tool” updated March 2014 assessed the risk of bias for animal studies (Table 3). The bias assessment for randomized controlled trials was performed using “revised Cochrane risk of bias tool for randomized trials (RoB2)” version of 22 August 2019 (Table 4).

**Table 3:** Systemic review centre for laboratory animal experimentation (SYRCLE) RoB tool

Author Name	Selection Bias	Performance Bias	Detection Bias	Attrition Bias	Reporting Bias	Other	Overall Bias
Fiorellini JP *et al.*, 2001	Moderate	Low	Low	Low	Low	Low	Low
Matin K *et al.* 2003	Low	Moderate	Moderate	Low	High	Low	Moderate
Smeeds R *et al.*, 2009	Low	Low	Low	Moderate	Low	Low	Low
Jaebum Lee, *et al.*, 2010	Low	Low	Low	Low	Low	Low	Low
Huh, *et al.*, 2010	Low	Low	Low	Low	Moderate	Low	Low
Lu SX, *et al.*, 2013	Low	Low	Low	Low	Low	Low	Low
Chang, *et al.*, 2017	Low	Low	Low	Low	Low	Low	Low
Schom *et al.*, 2017	Low	Low	Low	Low	Low	Low	Low
Sun YK, *et al.*, 2018	Low	Low	Low	Low	Low	Low	Low
Lyu HZ *et al.*, 2020	Low	Low	Low	Moderate	Low	Low	Low
Tan MH *et al.*, 2020	Low	Low	Low	Low	Moderate	Low	Low
Chao YL *et al.*, 2021	Low	Low	Low	Low	Low	Moderate	Low
Chao YL *et al.*, 2021	Low	Low	Low	Low	Low	Moderate	Low

**Table 4:** Revised Cochrane risk of bias tool for randomised trials (Rob2)

Author Name	Randomisation	Deviation from Intended Intervention	Missing Data	Measuring Outcomes	Selection of Reported Result	Overall Risk of Bias
Jung RE, *et al.*, 2003	Some Concerns	Low	Some Concerns	Low	Low	Low
Jung RE, *et al.*, 2008	Some Concerns	Low	Some Concerns	Low	Low	Low
Ragheb, *et al.*, 2017	Moderate	Low	Moderate	Moderate	Moderate	Moderate

### Summary measures and data synthesis

The data were summarised based on the type of samples (animal or human), the number of samples and implants, comparisons, follow-up months, type of carrier, the concentration of BMP, the methods of investigation, and outcome measured (Table 2). The outcome of the hypothesis; osseointegration around the dental implant was summarised qualitatively based on the type of bone, mineralization, bone density, bone height, bone-implant contact, and implant stability. Since there was no common outcome or measurement between the collected articles, quantitative measurement was not performed. The inter-rater reliability and kappa statistics were performed for agreement between the authors for the eligibility and inclusion section. The inter-rater reliability for FB and SK, SK and AK, AK and FB were 92.64%, 94.34%, and 94.34% respectively. The inter-rater reliability between the three authors was 93.71%. The kappa analysis of agreement between FB and SK, SK and AK, AK and FB were 0.81, 0.84, and 0.84 respectively suggesting almost perfect agreement.

## Results

### Study selection

**Figure 1:**
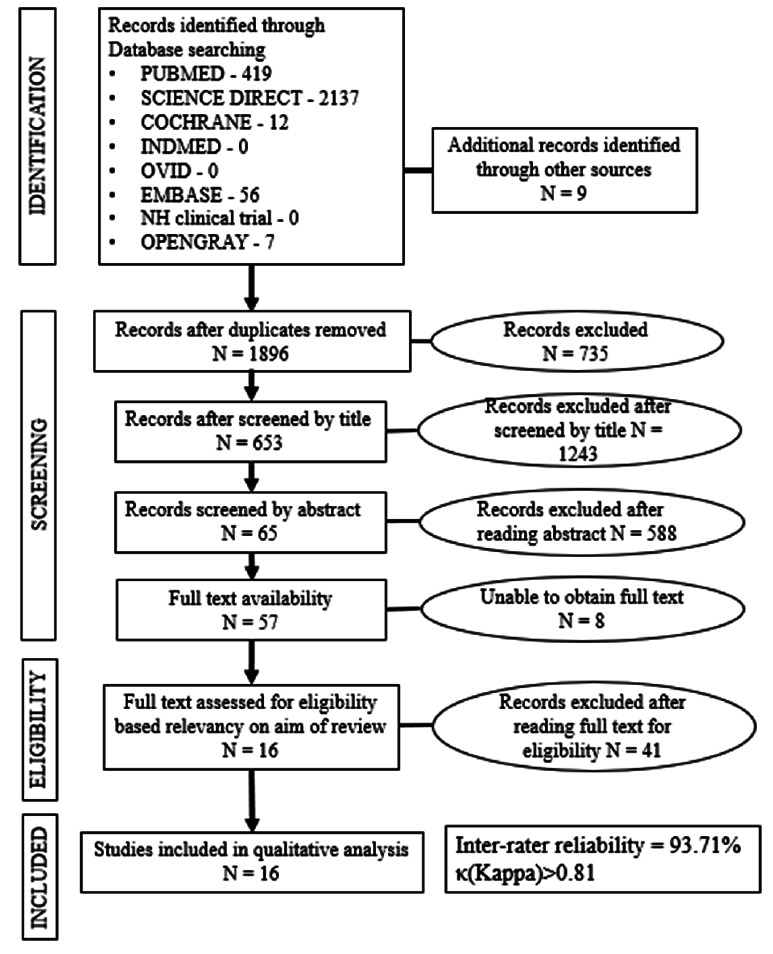
Systematic analysis of literature search

The search yielded 2631 articles (includes 419 Pubmed, 2137 Science Direct, 12 Cochrane, 56 Embase indexed articles, and also included 7 Opengray literature). 735 duplicate articles were filtered out and the remaining 1876 articles were further analysed by the title and the abstract to check their relevancy to the selected hypothesis. This yielded 57 articles, which was further filtered for full-text availability. We found that 16 articles were suitable for qualitative analysis based on the search criteria at the end of structured literature search (Figure 1). The meta-analysis was not performed due to heterogenicity between the selected articles on comparison of the outcome measure, type of carrier and concentration of BMP.

### Study characteristics

Based on the study hypothesis; 13 experimental comparative study designs on the animal models, and 3 randomized controlled trial study designs on the human model were selected. The number of implants placed in the oral cavities of animal models was 341 (mean of 31±22.04) with a mean of 32.66±20.68 in 42 canine models across the 6 articles, mean of 24±11.31 in 24 rat models across 2 articles, and mean of 32.33±35.16 in 22 minipigs model across 3 articles. The number of implants in RCT was 88 (mean of 29±8.66) in 32 (mean of 10.67±0.58) human participants across the three articles. The data extracted were tabulated in Table 2.

### Qualitative Synthesis of results

#### Carriers and Concentration of BMP

In the animal model, the delivery of rhBMP-2 around dental implants varied from calcium phosphate derivative, collagen base and poly (D,L-lactide-coglycolic acid) (PLGA) of which the former being the most common carrier. Jaebum Lee *et al.* [ 26 ] and Huh *et al.* [ 27 ] coated BMP directly on the implant surface without using any carrier. Jaebum Lee *et al.*, [ 27 ] coated 30 µg of rhBMP-2 by either soak or coronal loading and air-dried the implant surface. Huh *et al.* [ 28 ], compared two different lower concentrations of 10µg or 20µg of rhBMP-2 coated on implant surface by freeze-drying at -40ºC followed by vacuum drying at +20ºC.

Sustainable action of BMP was achieved by various authors with the use of a suitable delivery vehicle. Fiorelleni *et al.* [ 29 ] and Matin *et al.* [ 30 ] utilized methyl cellulose gel and PLGA coated gelatin sponge respectively, however, the quantity of rhBMP-2 dispersed on the peri-implant region was not specified. Various authors utilized hydroxyapatite/tricalcium phosphate (HA/TCP) carrier that could withstand functional load around implants and had an osteoconductive potential to deliver BMP [ 25 , 31 - 35 ]. Among the reviewed articles, Lu SX *et al.* [ 25 ], utilized a high concentration of 0.8 mg (0.4mg/ml) of BMP on HA/β-TCP (15:85%) in collaboration with the bio-ceramic bulking agent as a carrier to provide a compression-resistant matrix, and compared with 0.2 mg/ml of BMP in absorbable collagen sponges (ACS) carrier. Lyu *et al.*, [ 31 ] utilized 300 µg of rhBMP-2 in HA/β-TCP microspheres, while Chao *et al.*, [ 34 ] evaluated as low as 5, 20, 50µg of rhBMP-2 in HA/β-TCP (60:40 ratio) with collagen composite. Tan *et al.* [ 33 ] utilized biphasic calcium phosphate (BCP) with a HA/TCP ratio of 62/38 for carrying 0.43mg of BMP-2, however, the total quantity varied according to the defect size. Sun YK *et al.* [ 36 ] utlized a ACS reinfo-rced with hydroxyapatite as carriers in the concentration of 100µg (0.5mg/ml) BMP. Also, the efficacy of osteoconductive carriers; cryogel bone substitute comprising gelatin-HA/β-TCP with 0.16µg/µl of BMP and gelatin/ HA/β-TCP added to PLGA microspheres with 0.21µg of BMP were compared [ 37 ]. Insoluble collagenous bone matrix (ICBM) with vascular endothelial factor (18.4µg) (VEGF) was used to carry 138µg of BMP-2 [ 32 ]. The lowest concentration of 0.2 µg was tried in an animal study with a calcium phosphate carrier [ 38 ]. In human studies, the BMP carriers used were xenogeneic bone grafts, collagen and HA/ TCP with 0.5mg/ml, 1.5mg/ml, 0.25mg/ml of BMP respectively [ 22 , 39 - 40 ].

### Measuring outcome(osseointegration) based on concentration and carriers

The evidence of the efficiency of carriers and varied concentration were available only in animal experiments. Implant stability with a concentration of 10 and 20µg had values of 8 and 11.5 without the use of carriers [ 28 ], whereas an increase of concentration of 20µg and 50µg with HA/βTCP/collagen carrier had established a value of more than 75 [ 34 - 35 ]. Use of concentration less than 20 µg by Smeets *et al.* [ 38 ], (0.3µg), Huh *et al.* [ 28 ], (10, 20µg) and Chao *et al.*[ 34 ], (5, 20, 50µg) did not show significant improvement in osteoconductive potential compared to the control site, wherein the control site was the carrier. The concentration of BMP used in the reviewed articles varied between the range 0.215µg to 0.8mg and the outcome of the studies showed improved bone formation around implants despite the concentration, however, a significant effect was observed when concentration was above 20µg.

The bone density for the coronal and soak-loaded implants were 38 % and 34% respectively without the use of carriers [ 28 ], whereas the density of the peri-implant region with carrier ICBM and ICBM with VEGF increased to 46.1% and 65% respectively [ 2 ], signifying the importance of carrier. The bone-implant contact values without carriers averaged 25.0±3.8% and 31.2±3.3% for the soak loaded and coronal loaded implant [ 26 ], while with the carriers such as ICBM was 38 %, ICBM-VEGF was 49% while with biphasic calcium phosphate and HA/βTCP/Collagen increased to 82% and 60% respectively [ 32 - 34 ], signifying the efficiency of HA/TCP carrier. The bone remodelling was 0.06 mm/ day with the BMP group, while it was 0.01mm/day in the non-BMP group [ 29 ]. The mean bone height and the area were 3.4±0.2mm to 3.5±0.4mm and 2.6±0.4 mm2 to 2.5±0.7mm2 respectively for non-carrier BMP with more lamellar bone in the coronal loaded implant [ 26 ]. Schorn *et al.*, [ 32 ] revealed that with the use of carrier ICBM, the effect of BMP did not show significant change with vertical bone gain until 4 weeks but at 12 weeks the bone gain was achieved at 5.7mm compared to a non-BMP group of 3.1mm. Fiorellini *et al.* [ 29 ], observed a gain of 1.30±.39mm compared with 0.12± .18mm for a non-BMP group in methycellulose gel base. Whereas, with calcium phosphate carrier the bone gain was minimal with 2.37±.66mm with BMP and 2.25 ±.67mm without BMP [ 38 ]. Two animal experiments compared different carrier systems in promoting the efficiency of BMP. Lu SX *et al.* [ 25 ] revealed that the bone exhibited significantly increased area (20±-0.9 vs 12±2.6mm2) and density (24±1.4% vs.15±2.0%) around the rhBMP-2/CRM impregnated implant threads when compared with rhBMP-2/ACS. Chang *et al.* [ 37 ] stated that the gelatin/HA/b-TCP with PLGA microspheres showed generalised osteogenesis and increased mineralisation (51.6) in scanning electron microscope, when compared with gelatin/HA/b-TCP loaded BMP (mineralisation 20.3). The use of ACS as a carrier with rhBMP-2 showed seroma formation around implant when compared with HA/TCP carrier but the seroma decreased from 93% to 33% around the implants in 4 to 8 weeks [ 25 ]. The review of the studies revealed that the HA/TCP combination improved implant Osseo-integrative property of BMP than collagen sponge (ACS) carriers, which was evident through improved bone density, less seroma formation and ability to withstand functional load [ 25 , 31 , 33 - 35 , 37 , 40 ]. The reinforcement of hydroxyapatite carrier enhanced the structural strength of rh-BMP-2/ACS combination [ 25 ]. In addition, histomorphometry analysis proved that the fixation of collagen sponges using pins prevented the dislodgment of a carrier that improved the delivery of BMP at the localized site. The quantity of new bone formation, residual bone substitute, and non-mineralized tissue was 37.03%, 9.51%, 53.46% respectively compared to the group without pins to be 4.87%, 1.31% and 87.16 % respectively [ 36].

### Measuring outcome (osseointegration) based on BMP-2 vs non-BMP-2

The animal experiments revealed the efficiency of the BMP group compared with a non-BMP group. Chang *et al.* [ 37 ] revealed the absence of osteogenesis and reduced mineralization in the non-BMP group at 4 weeks. Lyu *et al.* [ 31 ], had also observed an insignificant increase in bone gain at 4 weeks, while Schorn *et al.* [ 32 ] and Chao *et al.* [ 34 ], also observed the same; they revealed maximum gain with BMP was achieved at 12 weeks and 8 weeks respectively, signifying the time of evaluation for BMP effect. The total augmented bone volume was also lower in the non-BMP group (2.75 mm3) compared with the collagen membrane reinforced BMP group (4.27mm3) [ 36 ]. Use of collagenous bone matrix also showed an increase of 42.1% compared to 2.1% between BMP and non-BMP groups respectively [ 32 ]. Whereas with HA/βTCP/Collagen increase in bone volume density was insignificant, that varied between 50-60% with the highest percent for the BMP group [ 34 ]. 

Similar trends were also seen in human studies [ 22 , 39 ]. Jung *et al.* [ 22 ] stated that the new bone formation with rhBMP-2 using xenogeneic bone substitute carrier was a mixed type including randomly arranged fibres of woven bone and the parallel fibre orientation of lamellar bone. This comparative analysis between the xenogeneic bone substitute with rhBMP-2 (test) and without rhBMP-2 (control) revealed that the average bone density was 37% and 30% respectively and mineralized bone (lamellar) were 76% and 56%, respectively in the peri-implant region [ 22 ]. The soft tissue assessment at 3-year follow-up showed less mean probing depths of 2.8mm (buccal sites) to 3.9mm (proximal sites) for the test implant, while 3.1mm (buccal sites) to 4.3mm (proximal sites) for the control implants [ 39 ]. However, comparison of BMP-2 with non-BMP-2 loaded implant revealed varied results depending on the time of analysis in the human model. 

Ragheb *et al.* [ 40 ] revealed that the non-BMP coated titanium implant group (control) had shown higher mean values of bone loss (0.43±0.3mm in control vs 0.39±0.33mm in test) and lower implant stability values (66.96±2.72 in control versus 67.87±2.23 in test) than the BMP coated titanium implant group (test) with a sta-tistically insignificant result at 1-year follow-up. Moreover, a statistically significant change in bone quality between the non-rhBMP-2 and rhBMP-2 groups was observed at 6 months, became statistically insignificant at 5 years, with only a mean change of 0.2mm [ 22 , 36 ]. The carriers and concentration in each of the studies differed and hence meta-analysis was not conducted in this systematic review for quantitative synthesis of the result. 

## Discussion

BMP-2, an osteo-inductive protein, has the ability to improve bone formation with a property similar to autogenous bone grafting [ 41 - 42 ]. For effective cellular ingrowth and stabilization at the grafted site, the osteo-inductive proteins require a delivery system or a carrier [ 24 ]. The combination of an osteoconductive carrier such as xenogeneic bone substitute with an osteo-inductive protein was effective in improving bone regeneration [ 22 , 39 ]. However, xenogenic bone substitute are antigenically dissimilar to human cells and can induce an immunogenic response [ 43 ]. The biomaterials such as collagen sponges, hydroxyapatite, fibrin, alginate, hyaluronic acid, and synthetic polymers were tried in both orthopaedic and maxillofacial bone augmentation [ 44 - 47 ]. Though collagen is neither osteoconductive nor can withstand the functional load, it is considered one of the best-described carrier materials for the growth factor [ 48 ]. 

On critical evaluation between the studies, collagen membrane was frequently used as a carrier for BMP. The review revealed that the collagen membrane as a carrier enhanced the osteo-inductivity of BMP-2 in the peri-implant region because of its ability to confine the growth factor in close proximity with the periosteum [ 22 , 32 , 36 , 39 ]. Fixation of collagen membrane with pins prevented mechanical failure, improved its stabilization and the efficiency of BMP-2 [ 36 ]. The use of carriers namely HA/TCP with bio ceramic bulking agent withstood the functional load and promoted the bone regeneration comparable to collagen sponge [ 25 , 37 ]. HA/TCP used in varying ratios modified as bicalcium phosphate was proved to improve the bioactivity and mimics as the natural bone without immunogenic response [ 33 ]. HA/βTCP without BMP and with BMP did not show a significant difference in their bioactivity until 4 weeks in the majority of the research, whereas effective change was observed after 8 weeks [ 32 - 35 ]. Cottam *et al.*[ 27 ], stated that the carriers were apparently osteoconductive agents and have the probability of inducing infection but the incorporated BMP suppressed the infection. During the initial phase of healing, the seroma (fluid-like collection) formation was observed around the implant coated with BMP, and carriers were evaluated for the possible causative factor in seroma formation [ 25 - 26 ]. Lu *et al.* [ 25 ] observed that the formation of seroma was less in HA/TCP carrier compared to the collagen sponge. However, the literature reveals that seroma is a dose-dependent sequellae of rhBMP-2-induced bone formation despite the presence of the carrier [ 26 , 45 , 49 ]. The qualitative analysis revealed the HA/TCP was better than collagen to withstand the functional load, reduced seroma formation and comparable to collagen in bone formation.

The BMP concentration available in the human bone was observed to be 1 µg/g of human bone matrix [ 13 ], but the quantity required for bone regeneration was much higher. The concentration of BMP used in the reviewed studies varied from 0.215 µg to 0.8 mg around the dental implant. Lee *et al.* reported that bone inductive potential of higher concentration of 20 µg BMP did not increase osseointegration compared to a lower concentration of 10 µg BMP on implant surface at 8 weeks without the use of carriers [ 26 , 28 ]. In addition, the researchers who worked independently with much lesser concentration at microgram level (.215µg) also proved the effectiveness of BMP in bone regeneration with the use of carrier HA/βTCP in PLGA [ 37 ]. In contrast researchers with calcium phosphate as a carrier did not find a significant difference at a lower concentration of 2µg of BMP [ 38 ]. Chao *et al.* [ 34 ], claimed that between 2, 20 and 50µg in HA/βTCP/ Collagen, 50µg of BMP-2 significantly increased the osseointegration. Wikesjö *et al.* [ 50 ] reported that a high concentration created a radiolucent area around an implant that did not affect the bone formation. They also stated that the immature bone was abundant with higher concentration while the mature bone was observed with lower concentration [ 50 ]. Though rhBMP-2 accelerated the mineralization and maturation of new bone, reports of displacement of implants with higher concentrations cannot be neglected [ 49 - 50 ]. Cowan *et al.* [ 51 ] suggested the concentration of BMP-2 can be as low as 30ng/mm3 to 240ng/mm3 with the porous PLGA scaffold in bone defects. While Hao-Chieh Chang *et al.* [ 37 ] used as low as 640ng/mm3 of rhBMP-2 in gelatin/HA/β-TCP cryogel composite to promote osteogenesis in vivo around the dental implant with effective osteogenesis and bone mineralization. Hence, the optimal concentration of rhBMP-2 for successful osseointegration of dental implants also depends on the type of carrier to provide sustainable release.The sustained release of BMP at a minimal concentration in dental implants was achieved with coaxial electrohydrodynamic atomization technique in an animal experiment [ 37 ]. The literature revealed that the coaxial electrohydrodynamic atomization technique controlled the release of BMP-2 by sustained PLGA degradation [ 52 - 53 ]. It incorporates BMP-2 and bovine serum albumin stabilizer in a hydrophilic core that reduced contact with the organic solvent in PLGA shell solution [ 37 ]. The high viscosity nature of the bovine serum albumin stabilizer solution more securely entrapped the BMP-2 into the microspheres, leading to its sustained release [ 31 , 37 ]. The slow diffusion reduced the concentration of rhBMP-2 required within the polymer to enhance the regeneration of bone.

The comparison of BMP-2 with non-BMP-2 implants sites revealed contradictory results in the enhancement of bone formation based on the period of evaluation. BMP did not achieve significant changes in outcome when the evaluation period was more than 6 months for human experiments [ 39 - 40 ]. In addition, the animal experiments that evaluated the efficacy of BMP for less than 4 weeks did not show significant changes denoting the effective bioactivity of BMP initiated after 1 month of delivery. Though implant stability measurement is an indirect measure of osseointegration, the reviewed articles used a less invasive and reliable device, the Ostell Mentor. The qualitative analysis revealed increased stability in presence of BMP-2 compared to the non-BMP-2 group. Enhanced bone regeneration during the initial stage of healing could be due to the rapid release of BMP-2 in the surrounding environment that promoted the signalling pathway by stimulating osteoprogenitor cells in the grafted site [ 54 ]. Moreover, the method of evaluation in the research articles (animal experiment) was histological or histochemical analysis during the initial period of healing, which revealed the microscopic changes in the bone due to BMP. However, the BMP-2 that initially enhanced the extracellular matrix with the formation of woven trabecular bone and then remodelled to the lamellar bone, had taken a longer period for completing mineralization [ 20 ]. This could be the reason behind an insignificant change in radiographic evaluation taken after 6 months of implant placement.

This systematic review enumerated the outcome of the BMP around the dental implant as an additive material incorporated during the surgical phase based on its concentration, type of carrier, and the time of assessment. The trends in animal researches were also searched in human studies with the highest level of evidence. The review also revealed that the updated research done in animal model is still lacking in human research (randomised controlled trial). A limitation of the review was inclusion of both human and animal study designs due to the availability of limited and heterogenous research articles based on focus question. Moreover, the efficacy of BMP as an alternative to graft material around the dental implant in human participants was minimal in randomised controlled trial. The qualitative analysis revealed voids in this field of human research, and we have put forth a few scopes for future research with BMP as follows. First, long-term research on the human model should assess the osseointegration of dental implants based on different carrier systems and varied concentrations of BMP-2. Second, research on biodegradable carriers like PLGA that provides sustained release of BMP without mechanical failure on a human model should be performed. Third, the possible role of BMP in the prevention of osteoclastic activity around dental implants using immunohistochemistry and bone scintigraphy should be investigated. A randomized control trial to evaluate the above criteria would be more beneficial to the clinician and researchers. 

## Conclusion

The conclusion of this systematic review is based on minimal literature availability and further researches on analysing sustained, local delivery of BMP as an alternative to bone graft material with long-term follow-up around the dental implant in the human model would add strength to the available literature evidence. Qualitative analysis revealed BMP was effective in accelerating bone growth during the initial stages of healing in the human model, and with the carriers namely; collagen sponge, HA/βTCP, HA/βTCP/Collagen, biphasic calcium phosphate, ICBM and the PLGA in HA/TCP. The osteo- inductive potential of rhBMP-2 is enhanced in the presence of a compatible carrier material at even lower concentrations. Among the carriers, animal experiments revealed HA/TCP had higher strength in withstanding functional load at a lower concentration of 50µg, while PLGA microspheres with gelatin/HA/TCP carrier had better osteogenic and mineralisation potential when delivering BMP of lower concentration of .215µg around the dental implant. 

## Conflict of Interest

The authors declare that they have no conflict of interest
